# European Reference networks for rare diseases: what is the conceptual framework?

**DOI:** 10.1186/s13023-017-0676-3

**Published:** 2017-08-07

**Authors:** Véronique Héon-Klin

**Affiliations:** Bundesministerium für Gesundheit, Rochusstr. 1, 53123 Bonn, Germany

**Keywords:** European Reference networks, Health networks, Knowledge transfer and expertise, Cross-border networks in health care, Connectivism, Digitisation, Connective action

## Abstract

With the Cross-Border Healthcare Directive (2011/24/EU) a mandatory framework was established to foster cooperation on a voluntary basis, within European Reference Networks (ERNs). These networks are composed of centres and healthcare providers. The exchange of knowledge is a central issue in this context. A detailed literature survey was carried out to determine the most important factors affecting information and knowledge exchange, as well as learning, in networks and how this can be supported. New communication technologies are identified as key tools for the European Reference Networks (ERN). This study recommends the elaboration of a systematic knowledge use and knowledge generation plan. The data of this study suggests that the future ERNs will mediate the adoption of the digitised and networked information society in medical practice.

## Background

In February 2017, 24 European Reference Networks (ERNs) were established in a European legal framework, of which 23 are dedicated to rare or low prevalence complex diseases or conditions (Table 1). More than 300 hospitals and 900 highly specialised teams are participating in the approved ERN. These networks were launched in March 2017 in a context of uncertainty about the way they will operate, due to lack of previous experiences. If European networks had been funded in the past, they were mostly financed for activities dedicated to research and to data collection, not for care. Moreover, they were established between individuals and not between institutions.

**Table 1:** List of the established European Reference Networks (ERN)

1. BOND	European Reference Network on Rare Bone Disorders
2. CRANIO	European Reference Network on Rare craniofacial anomalies and ENT disorders
3. Endo-ERN	European Reference Network on Rare Endocrine Conditions
4. EpiCARE	European Reference Network on Rare and Complex Epilepsies
b5. ERKNet	European Rare Kidney Diseases Reference Network
6. ERN-RND	European Reference Network on Rare Neurological Diseases
7. ERNICA	European Reference Network on Rare inherited and congenital anomalies
8. ERN-LUNG	European Reference Network on Rare Respiratory Diseases
9. ERN-Skin	European Reference Network on Rare and Undiagnosed Skin Disorders
10. EURACAN	European Reference Network on Rare Adult Cancers (solid tumors)
11. EuroBloodNet	European Reference Network on Rare Hematological Diseases
12. EURO-NMD	European Reference Network for Rare Neuromuscular Diseases
13. ERN-EYE	European Reference Network on Rare Eye Diseases
14. ERN GENTURIS	European Reference Network on GENetic TUmour RIsk Syndromes
15. GUARD-HEART	Gateway to Uncommon And Rare Diseases of the HEART
16. ITHACA	European Reference Network on Rare Congenital Malformations and Rare Intellectual Disability
17. MetabERN	European Reference Network for Rare Hereditary Metabolic Disorders
18. PaedCan-ERN	European Reference Network for Paediatric Cancer (haemato-oncology)
19. RARE-LIVER	European Reference Network on Rare Hepatological Diseases
20. ReCONNET	Rare Connective Tissue and Musculoskeletal Diseases Network
21. RITA	Rare Immunodeficiency, Autoinflammatory and Autoimmune Diseases Network
22. TRANSCHILD	European Reference Network on Transplantation in Children (incl. HSCT, heart, kidney, liver, intestinal, lung and multiorgan)
23. VASCern	European Reference Network on Rare Multisystemic Vascular Diseases
24 EUROGEN	European Reference Network on Rare Urogenital Diseases

To shade light on what the newly established ERNs can, or should, put into place to fulfil their obligations, this article reviews both the legal framework establishing a potential collaboration between healthcare institutions and the conceptual framework of network activities observed in the healthcare field in general. The purpose is to underline the principles which should better be respected to ensure that the ERNs deliver the expected added-value, and finally recommend possible instruments and tools which could promote, nationally and at the European level, the exchange of knowledge and information within and between ERNs, and to support the establishment of collaborative network structures nationally.

### The concept of ERNs

With the directive 2011/24/EU [1] of the European Parliament and of the Council on the application of patients’ rights in cross-border healthcare (cross-border directive) a mandatory base was established to cooperate in a more structured way and on a voluntary base, in line with the subsidiarity principle, in highly specialised healthcare.

In this Directive, and the ensuing Commission Delegated and Implementing Decisions, the establishment of European Reference Networks (ERN) between health care providers and centres of expertise (CE) was agreed upon. An ERN is composed of at least ten healthcare providers from at least eight different Member States (MS). The overarching aim is to facilitate access to highly specialised health care for patients requiring a concentration of resources or expertise. Because of the low number of patients and medical experts, the lack of knowledge in diagnostics and therapeutic options, as well as because of limited resources, no single MS in isolation will be able to provide access to the best possible healthcare in all areas of highly specialised health care for the residents of their country concerned by rare diseases. The criteria and conditions, which CE and health care providers have to fulfil to become part of an ERN, are determined in the Commission Delegated Decision (2014/286/EU) [2]. The criteria of the implementation, evaluation and the facilitation for sharing knowledge of the ERN are determined in the Commission Implementing Decision (2014/287/EU) [3] (Table 2).

**Table 2:** Relevant criteria for European Reference Networks (ERN)

Themes in the Operational Criteria		
	Network	Healthcare Providers
Themes	• Establishment of a European Reference Network • Highly Specialised Healthcare • Governance and Coordination • Patient Care • Multidisciplinary Approach • Good Practice, Outcome Measures, and Quality Control • Contribution to Research • Continuous Education, Training, and Development • Networking and Collaboration	*General Criteria:* • Patient Empowerment and Patient-Centred Care • Organisation, Management, andBusiness Continuity • Research, Education and Training • Expertise, Information Systems, and e-Health Tools • Quality and Safety *Specific Criteria:* • Competence, Experience andOutcomes of Care • Human Resources • Organisation of Patient Care • Facilities and Equipment
Assessment	Each criterion is rated once for the overall Network.	Each criterion is rated individually for each Healthcare Provider within the Network.

(Published in the ERN Assessment Manual for Applicants and dowloaded on the 6th of march 2017 http://ec.europa.eu/research/participants/data/ref/other_eu_prog/hp/guide/pse/hp-asses-manual-ern-descr-process_en.pdf)

**The most important criteria for knowledge and information sharing from the COMMISSION DELEGATED DECISION of 10 March 2014 setting out criteria and conditions that European Reference Networks and healthcare providers wishing to join a European Reference Network must fulfil (2014/286/EU)**

(4) To fulfil the requirement set out in point (iii) of Article 12(4)(a) of Directive 2011/24/EU (‘offer a high level of expertise and have the capacity to produce good practice guidelines and to implement outcome measures and quality control’), the Networks must:

(a) exchange, gather and disseminate knowledge, evidence and expertise within and outsidethe Network, in particular on the different alternatives, therapeutic options and best practices with regard to the provision of services and the treatments available for each particular disease or condition;

With the Treaty of Maastricht the European Union (EU) has created a common, free and competitive internal market stipulating the Free Movement of persons and an EU-citizenship [4, 5]. At the European level, three types of EU-interventions are distinguished: market-building policies (e. g. trade, competition, internal market, common commercial policy related rules); market-correcting policies (e. g. EU Structural Funds, the Common Agricultural Policy) and market-cushioning policies (e. g. environmental policies, occupational health and safety).

The market-building policies, with the creation of an integrated internal market, have had the largest impact on the health care policies of the EU and the Member States (MS). In the health care field the EU can only act if the policies of the MS are insufficient and when political objectives are better achieved at community level (principle of subsidiarity). Because of this, the primary competence of the MS for its health care system and its social protection systems, the payment of benefit entitlements of insured persons is enshrined in the social protection system of the state of employment. In the Treaty of Lisbon, health protection is defined as a cross-sectional task in all different policy fields. Support for cross-border healthcare between MS is stated as a special strategic focus area of the EU, whose goal is to increase the complementarity of health care services in the cross-border regions. This takes place in general through bi- and trilateral health care cooperation. In contrast to this type of cooperation, the collaboration envisaged in the context of ERNs is not limited to border regions of a MS, but encompasses the entire territory of the MS (Table 3).

**Table 3:** Relevant policies to patient mobility

Regulation (EC) No 883/2004 of the European parliament and of the council of 29 April 2004 on the coordination of social security systems
Directive 2011/24/EU of the European parliament and of the council of 9 March 2011 on the application of patients’ rights in cross-border healthcare
Commission delegated decision of 10 March 2014 setting out criteria and conditions that European Reference Networks and healthcare providers wishing to join a European Reference Network must fulfil (2014/286/EU)
Commission implementing decision of 10 March 2014 setting out criteria for establishing and evaluating European Reference Networks and their Members and for facilitating the exchange of information and expertise on establishing and evaluating such Networks (2014/287/EU)

Patient mobility, within the EU, is based on two EU regulations: Regulation (EC) No 883/2004 [6] on the coordination of national social security systems and the Cross-Border Healthcare Directive 2011/24/EU, which guarantees free movement for all patients of the EU and the control of health care expenditures for MS. Any healthcare expenditure due to cross-border healthcare requires prior authorisation for care interventions with increased planning needs such as hospitalisations. This authorisation needs to be granted by national insurance providers. For outpatient treatments the total amount of reimbursement has to be in line with the expenses of a comparable national treatment (also taking into account some deductions from extra administrative costs). In all MS there are national regulations to balance or take countermeasures to maintain social sustainability (“The European social model in health”). There are no instruments for counter measures at European level.

This unresolved asymmetry between European competition regulations and the need for socially responsible and sustainable health care systems in the MS creates a complex area of differing and conflicting interests (which in turn determine the positions and actions of the different actors). With regard to ERNs, two key drivers can be identified: on the one hand medical experts and scientists (because of the existing interdependence of research and care in this highly specialised field [7]) and on the other hand patients and their families, including the patient organisations. These stakeholders are supported in their common cause to improve access to highly specialised care by the European Commission.

Some MS however have reservations concerning the establishment of the ERNs, but nevertheless 25 EU Member States plus Norway decided to authorise the participation of their health care providers in the call for ERNs. At present, individual cross-border patient mobility takes place on a small scale. According to the results of Eurobarometer in May 2015, entitled “Patients’ rights in cross-border healthcare in the European Union”, only 2% of EU citizens took the decision to receive treatment abroad. So the consequences and the potential financial burden associated with the establishment of the ERNs cannot currently be assessed by MS [8]. Therefore, it will be indispensable for the political and medical success of the ERNs to find a balance between the free internal market and “the European social model in health”. At the same time, there remains a risk that the desperate hopes of patients will be dashed. Due to the heterogeneity of healthcare systems with different financial opportunities and resources, the prior authorisation system (the prerequisite for treatment within an ERN) might increasingly be perceived as a problem for patients’ access. Here, particularly, the European and national patient organisations will play a bridge-building role in the future to improve patients’ awareness and understanding of these regulations.

### ERN for rare diseases

Directive 2011/24/EU acknowledges explicitly, in Art.12 and Art 13., the unique potential of ERNs for the rare disease (RD) field. The EUCERD adopted Recommendations on Rare Disease European Reference Networks on 31 January 2013 [9], and an addendum to these recommendations on 10 June 2015 [10].

The primary purpose of these Recommendations was to “*help focus on the specificities of rare diseases and the criteria for the establishment and evaluation of ERNs in the field of rare diseases*”. The purpose was to support as far as possible the use of information and communication technologies to ensure remote access to specialised healthcare when needed, and to organise the mobility of expertise and knowledge in order to facilitate patients’ care close to their homes, while providing solutions for all patients, whatever their disease.

### The concepts of information and knowledge exchange

Without prejudice to the importance of the above issues, the relevant question is how information and knowledge exchange, as well as learning, can be structured and organised within ERNs. These concepts play a central role in the success of networks. The declared objective of an ERN is, that at first scientific findings and specific expertise are exchanged within and between – cross-border – networks, with a view to creating access to highly specialised healthcare at local, regional and national level. This refers in particular to different therapeutic options and good practices concerning a single rare disease or a highly complex health problem (see Annex I, point 4a of the Delegated Decision). It is tacitly assumed that knowledge will be exchanged across borders more easily (even automatically) via network structures [11]. In contrast, at present patients have to travel long distances for their care, often at great expense and under difficult conditions such as separation from their family, language barriers, foreign cultures, as well as many other factors [12].

Information and knowledge exchange in the framework of future ERNs represent a challenge in themselves considering the size of the ERNs, as well as the different cultures, languages and various health systems with heterogeneous administrative and regulatory requirements. Information- and knowledge exchange is foreseen in an area that is resource-intensive and, because of the interdependence of research and care, also very innovative. Therefore, it takes place in a highly competitive area in terms of professional development and scientific careers [13]. This complicates successful information- and knowledge exchange, which will be vital in ensuring the acceptance of these voluntary healthcare networks by all the different players.

Highly specialised knowledge, which necessitates a concentration of resources, is usually rare and certainly not automatically exchanged. Therefore the central question is, which factors influence information- and knowledge exchange as well as learning in networks and how these processes can be supported.

In social sciences, a network is defined as an association of a definite number of individuals connected through social relations (e. g. common interests, providing mutual support and information) of varying depth [14]. Godwin et al. [13] differentiates several types of networks according to the nature of links between the different institutions/organisations of a network (loose or tight, weak or strong, bound or unbound, formal or informal). They can be peer structures (networks composed of CE with a similar specialisation [“enclave structure”]); hub and spoke networks (one or more CEs nested in non-specialised providers (i.e. hierarchical networks)) or organic structures (networks of different kinds of health institutions [i.e. individual networks]), the links of which are less formalised and work together on a voluntary basis.

In the health care field most networks are hybrid, which means the networks are composed of all three types in a different mix. There exists some research on dissemination strategies on information in networks depending on their structure. The design of the dissemination strategies differs i.e. in enclave structures and hub and spoke structures depending on the amount of links between the network members, the concentration of information is within some central actors and depending on the information flow [15]. As most healthcare networks are hybrids and the ERN will be composed of different protagonists, new dissemination strategies will have to be developed. In networks communication, information and knowledge exchange, as well as learning, take place simultaneously and it is difficult to dissociate these two concepts. The Director General of the Directorate General for Health and Food Safety phrased it as follows “Knowledge represents the sharing of information in collaborative networks in settings of increasing complexity and uncertainty” [16].

In networks, two learning strategies exist: exploration as the experimental search for new competencies or knowledge (including blue sky research) and exploitation as the application and implementation of existing competence and knowledge [13]. Furthermore human knowledge is differentiated in tacit (know-how, mostly unwritten) and explicit knowledge (written, encoded rational knowledge). Tacit knowledge dominates under conditions of competition for internal promotion or the control of resources (e. g. academic and industrial research).

“The Knowledge-Creating Company” [17] is a relevant publication for this topic. In Asian cultures the generation of new knowledge generally develops in a spiral, iterative conversion process of tacit knowledge to explicit, documented knowledge (externalisation) at the level of a group/team. Personal beliefs (ideals) and formal, abstract ideas/models form a complementary, dynamic balance. The generation of knowledge results from the exchange of tacit and explicit knowledge in group processes of knowledge. These include practitioners who work on the ground and function as knowledge engineers/facilitators by converting the different forms of knowledge in such a way that knowledge officers such as top managers can pilot the entire knowledge generation process. In the Western tradition the main focus is on explicit knowledge. The conversion of implicit to explicit knowledge in healthcare can be supported by personal reports, case-books and clinical reports. Sir Muir Gray [18] defines knowledge as “information organised for action”. According to him 10% of healthcare organisations are defined by the structure of bureaucracy, 40% by the medical field/system and 50% by leadership. Therefore, the role of leadership [19] has the greatest impact on changing healthcare organisations and is the most important lever for the generation of knowledge and change [14, 15]. The number of hospital alliances in health care has significantly increased over the past years with the primary objective of pooling resources (sharing of equipment, expertise, costs, operations and human resources) [20]. However, the expected benefits were reaped in less than only 50% of the alliances examined. What seems to be important for the success is the capacity of networks to coordinate and maintain concerted efforts. This assumption is also confirmed by other publications from the UK National Health Service [21] and Lega [22].

Another important influencing factor in the area of information and knowledge exchange, as well as learning, is the durability of knowledge. New digital media and technologies have fundamentally altered this dimension of information and knowledge exchange [23], that is why the half-life of knowledge has decreased significantly. Learning (of valuable knowledge) has become a perpetual process, which no longer only activates the individual’s knowledge, but relies more and more on the capacity of the individual to connect and newly evaluate external human sources such as other experts and non-human sources of knowledge such as databases, also called nodes, in a constantly changing environment. The daily evaluation of connections and connectivity becomes more important than our current state of knowing/knowledge. The capacity to evaluate which sources of knowledge (nodes) should be linked together by a connected individual is, according to the learning theory of George Siemens [24], itself a learning process. The individual determines and cares for his own learning networks/nodes. Here the connections which are of different quality (loose/tight) become more important than the content. Crucially, a constant external and internal exchange between human and non-human sources occurs and evolves.

Some publications suggest that there is a shift to a more open and inclusive society - a society which is driven and influenced by information from the World Wide Web [24]. The dividing lines blur between “lay persons” and “experts”, between “individual” and organisation/institution, between “public” and “private”, between “virtual” and “real” as well as between “national, European and global” [25]. So, the roles and functions of the different players have changed, e.g. from the passive role of patients and their relatives to the active role of decision makers and “expert lay people” in the field of RD. For many RD there are no treatment protocols, medical guidelines and causal therapies. Patients and their families researching their own disease until a diagnosis is made (and beyond) have become a valuable, additional source of specific knowledge. Therefore, patient organisations should be given the role of acting as mediators between the different players in the multidisciplinary networks. For example, they have the opportunity to contribute to the knowledge generation via social media.

In addition to technically assisted networking, new digital phenomena like crowdsourcing arise, which are also developing in healthcare. For example, external and internal multidisciplinary experts are connected via a secure, web-based interface (crowdsourcing international expertise) in 26 different European countries and contribute to diagnosing rare genetic syndromes of multiple congenital anomalies [26]. Ranard et al. [27] call for the speedy development of standards and guidelines in this context. Also in patient networks first studies point out, that social media as Facebook, YouTube and Twitter are more frequently used than instructive data. Here, too, the phenomenon of crowdsourcing emerges, which should be closely investigated by scientific methods and analyses.

The importance of personal contacts for the exchange of tacit knowledge based on trust was emphasized above all. These processes can be reinforced by the exchange of personnel, placing staff on each other’s site, joint decision making bodies between organisations at the operational level, best practice exercises, development of standards for operations as well as common protocols and guidelines [13].

Hierarchical and managerial network structures hinder the ability to innovate and learn. Further obstacles to collaborations come from different professional disciplines, competing priorities, different legal obligations, incompatible cultures, personal rivalries and skills deficits.

The collaboration of the future ERN refers to the entire territory of MS, however in a very limited scope of highly specialised healthcare. There is the central premise that first medical expertise will cross the border and that only in a few cases (e. g. for highly specialised interventions, for diagnostic and therapeutic measures which are not available in the “home country”) the patient will travel. So in most of the cases the treatment of the patient occurs in the health system the patient belongs to. This way, the different administrative and legal framework conditions should not so much impact the overall process. The provision is made that the MS that are part of the Board of Member States – a steering committee - ensure that the activities at the European level adhere to national requirements and regulations of the MS. This board officially designates the ERN. As medical experts are used to exchanging knowledge with each other in English, especially in research in this highly specialised field of expertise, cultural and linguistic differences should not have such an impact. Further differences are of note: it is envisaged that cross-border communication takes place primarily via IT media such as videoconferences for which there is little experience. Also, there are a number of unresolved data protection issues and no existing sustainable financing mechanisms. Furthermore, IT-based medical services, like expert advice or remote diagnosis, are currently not refundable. They are neither included in national catalogues of benefits and services, nor does a “fund” exist for such services at European level. Currently common European standards of care are missing in the field of highly specialised healthcare. Each ERN will have to develop common standards (taking into account the national standards).

MS prioritise domestic solutions to cross-border solutions as the latter are seen as challenge for the health system [8]. Policy makers have few reasons and few tools for promoting cross-border collaboration for medical care. There are no standards and the solutions will be complex, context-dependent and specific. Therefore, MS are likely to take an indifferent or reluctant approach to cross-border collaboration. Interventions of the EC to support cross-border collaboration, such as funding of projects, are of limited duration and not always welcome, as they are perceived as interference with the national health systems (« Principle of subsidiarity »). Furthermore, a lack of sustainable funding is hindering the implementation of ERNs. An appropriate level of funding is an essential prerequisite for sharing knowledge and information (learning) in networks. National health systems normally do not include financial incentives for CE to allow them to provide cross-border clinical support. It is still unclear how cross-border coordinative services, beyond the services of the EU financed IT-platform, are going to be funded.

### Identified issues to be considered or addressed

Knowing the factors promoting or hindering information and knowledge exchange, as well as learning, among network members, enables recommendations to be established to ensure the success of this new form of healthcare organisation.

**Fig. 1:**
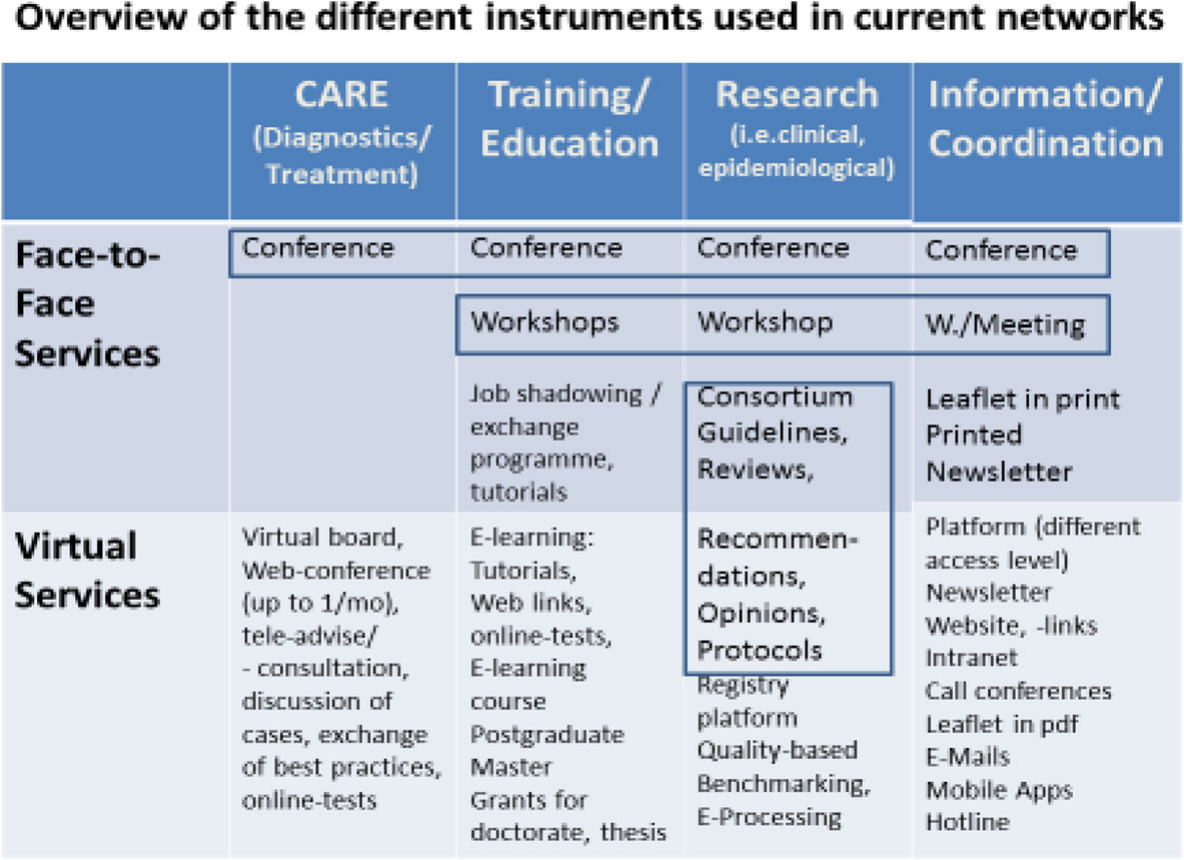
Overview of the different instruments used in current networks. Legend: Within the networks numerous instruments for communication and the exchange of knowledge are used. They can be devided into face-to-face and virtual instruments

Networks in highly specialised health care are hybrid structures composed of several types of networks with different levels of collaboration. There is normally a core of players, who work together based on contracts with clearly defined deliverables. This inner circle can be composed of complementary or similar highly specialised institutions of the same area as well as other stakeholders (e.g. laboratories, patient associations, medical societies). Other collaboration partners, based on statutes, common declarations without financial support, connect to this inner circle and participate in e. g. working groups of networks in the development of medical guidelines, treatment recommendations and protocols. The network coordinator plays a central role as motivator and enabler. He has to be a specialist in handling new media (see Fig. 1) and motivating the different network members by showing leadership with clear targets.

The half-life of knowledge has shortened so much that knowledge exchange and learning have become a process which, according to the theory of George Siemens [24], is not only based on the acquired knowledge of a person, but primarily relies on his capacity to connect various exterior, highly specific, human and non-human sources of knowledge, and to reassess these elements.

The European heterogeneity of health systems promotes creative knowledge generation in this highly innovative field. The generation of new knowledge (exploration) and the application of known knowledge (exploitation) take place in equal parts due to the interdependence of care and research, where tacit clinical experience merges gradually with explicit validated knowledge. The requirements for such concepts of knowledge generation and knowledge use in the four areas of medical practice could support the networks to think in a systematic way about what knowledge is generated where and how it can be used.

ERNs can be used as showcase to gain practical experiences with already developed instruments such as patient summaries and E-prescription through pooling the European and National Programmes for e-health in the area of highly specialised healthcare. This is also relevant for other areas such as data exchange, which remains a challenge because of data protection regulations and issues of interoperability. Solutions seem to be mostly specific and not generic.

Pilot e-health projects could be evaluated for their utility for the ERNs, such as the project of the Bertelsmann Foundation [28] to overcome likely language barriers. Interpreters could be consulted via web-based video solutions. In collaboration with the MS, sustainable solutions could be developed, e. g. through structural funds to finance virtual, cross-border, highly specialised care advice services.

A new culture of cooperation might develop at European level and it is expected that the expertise will slowly converge over time. Some research on the macroculture of networks suggests that concentrating on the way these networks produce their core artefacts such as their logo, common publications, collective products or services, will have the most influence on the formation and operation of the network [29]. Core artefacts can be more easily influenced than espoused values or basic underlying assumptions. But this research did not take into consideration the influence of digitisation or cross-border communication in networks. So further research is needed to discover how to stimulate changes in networks’ core practical activity. MS and the national players can support the ERN process through e-health projects, by introducing e-services in their national catalogues, by supporting integrated research and care centres and networking of the players.

Academic institutions might consider changing their performance and promotion systems in such a way that a culture of sharing and being a good team player develops, at the same time maintaining some level of beneficial competitiveness.

Successful ERNs will contribute to successful R&D, thanks to the interdependence of research and care in this area, to the development of treatment protocols utilising the knowledge of patients and experts. However it is important to protect the scientific integrity of the scientists/physicians and to secure the public acceptance and trust in these networks by clarifying the role of Industry as a partner of these ERNs. Therefore, an appropriate governance of the ERN activities is necessary [30].

Differences in national reimbursement schemes for diagnostics and treatments and het-erogeneity of the existing healthcare systems are going to remain a source of frustration for the patients that the ERNs will not be able to overcome.

However, the high degree of diversity of the different European health systems with their different financial capacities without common European guidelines might cause some disappointment as expectations are high. Patient associations will be a important players of the newly created ERNs.

Patients and patient associations can connect through the social media, generating practical knowledge and act as “mediator” between different players. They will have an important role as a “bridging” mediator, a capacity in which they should be empowered. Irrespective of the scientific progress in this area, particularly in the area of RD, there are patients for whom there is no causal treatment yet. Therefore, caution should be taken to avoid creating unrealistic expectations. Also, individual privacy rights have to be protected and there is a need to develop new approaches to palliative care.

## Conclusion

The best medical centres and faculties of Europe are connecting in 24 networks composed of nearly a thousand of centres of expertise or highly specialised healthcare providers (see Table 1). The newly established ERN will have a substantial impact on medical practice. In short, it marks the start of a new era in cross-border cooperation and in healthcare organisation.

ERNs represent the ideal structure for European cooperation and will not impede the overall functioning of the national healthcare systems as long as the focus is on information and knowledge exchange and as long as the patient is only treated in duly justified cases in the “foreign” system.

The main key challenge is to provide evidence of the added value of these networks for all players, in particular the MS. For the first time MS will not only cooperate with one or two MS on the basis of framework agreements and administrative arrangements, but with at least 8 MS whose health systems are different. We need to identify and to monitor indicators of relevance for the individual players, from the beginning, so that the process is assessed. This will be essential to convince stakeholders that the additional efforts involved in the evolution towards a new healthcare organisation, are justified, and to help take countermeasures if necessary.

In the years to come the boundaries between virtual/real, public/private, institutional/individual and expert/amateur are likely to blur. We should be prepared to face this change. The Cross-Border Healthcare Directive has established the necessary administrative and legal framework for new practices which will modify the role of healthcare providers. How this will affect their clinical practice and expertise is not known. How this will affect the career promotion system and the balance of competition/cooperation between institutions is uncertain. How the valuable practical knowledge of patients within the social media context will find acceptance and be used for the generation of knowledge in networks is unknown. How patient organisations will become sufficiently empowered remains unclear. Remote management should not affect negatively the need for empathy and individual privacy. The interplay between tacit and explicit knowledge will raise difficulties when defining common standards. Many questions remain unanswered at this point. The ERNs are an opportunity to explore these changes and turn them into assets for patients with unmet needs. If we succeed in promote knowledge exchange in such a way that patients will only be treated in duly justified cases in the “other” MS, then “the European social model in health” can be preserved under the current European treaties.

## Abbreviations

CE: Centers of Expertise
Cross-Border Healthcare Directive: Directive 2011/24/EU of the European Parliament and of the Council of 9 March 2011 on the application of patients’ rights in cross-border healthcare
EC: European Commission
ERN: European Reference Networks
EU: European Union
MS: Member States
RD: Rare Diseases

## References

[1] Directive 2011/24/EU of the European Parliament and of the Council of 9 March 2011 on the application of patients’ rights in cross-border healthcare. 2011. http://eur-lex.europa.eu/LexUriServ/LexUriServ.do?uri=OJ:L:2011:088:0045:0065:en:PDF. Accessed 15 June 2017.

[2] 2014/286/EU: Commission Delegated Decision of 10 March 2014 setting out criteria and conditions that European Reference Networks and healthcare providers wishing to join a European Reference Network must fulfil. 2014. http://eur-lex.europa.eu/legal-content/EN/TXT/PDF/?uri=CELEX:32014D0286&from=EN. Accessed 15 June 2017.

[3] 2014/287/EU: Commission Implementing Decision of 10 March 2014 setting out criteria for establishing and evaluating European Reference Networks and their Members and for facilitating the exchange of information and expertise on establishing and evaluating such Networks. 2014. http://eur-lex.europa.eu/legal-content/EN/TXT/PDF/?uri=CELEX:32014D0287&from=EN. Accessed 15 June 2017.

[4] Hennion S, Kaufmann O (Hrsg.). Unionsbürgerschaft und Patientenfreizügigkeit/Citoyenneté européenne et libre circulation des patients/EU citizenship and free movement of patients. Berlin Springer Verlag. 2014.

[5] Mossialos E, Permanand G, Baeten R, Hervey T (2010). Health systems governance in Europe: the role of the European Union law and policy.

[6] Regulation (EC) No 883/2004 of the European Parliament and of the Council of 29 April 2004 on the coordination of social security systems. 2004. http://eur-lex.europa.eu/LexUriServ/LexUriServ.do?uri=OJ:L:2004:166:0001:0123:en:PDF. Accessed 15 June 2017.

[7] Hannemann-Weber H, Kessel M, Schultz C (2012). Research performance of centers of expertise for rare diseases – the influence of network integration, internal resource access and operational experience. Health Policy.

[8] Glinos IA, Wismar M. Hospitals and Borders: Seven case studies on cross-border collaboration and health system interaction. Observatory Studies Series No. 31. European Observatory on Health System Policies. 2013. http://www.euro.who.int/__data/assets/pdf_file/0019/233515/e96935.pdf. Accessed 15 June 2017.

[9] European Union Committee of Experts on Rare Diseases (EUCERD) Recommendations to the European Commission and the Member States on European Reference Networks for Rare Diseases (RD ERNS). 2013. http://www.eucerd.eu/?post_type=document&p=2207. Accessed 28 May 2017.

[10] Commission Expert Group on Rare diseases. Addendum to EUCERD Recommendation of January 2013. http://ec.europa.eu/health//sites/health/files/rare_diseases/docs/20150610_erns_eucerdaddendum_en.pdf. 2013. Accessed 28 May 2017.

[11] Palm W, Glinos IA, Rechel B, Garel P, Busse R, Figueras J. Building European Reference Networks in Health Care: Exploring concepts and national practices in the European Union. Observatory Studies Series No. 28. European Observatory on Health System Policies. 2013. http://www.euro.who.int/__data/assets/pdf_file/0004/184738/e96805-final.pdf. Accessed 15 June 2017.

[12] Commission Report on the operation of Directive 2011/24/EU on the application of patients’ rights in cross-border health care. COM (2015) 421 final. 2011. http://ec.europa.eu/health//sites/health/files/cross_border_care/docs/2015_operation_report_dir201124eu_en.pdf. Accessed 15 June 2017.

[13] Godwin N, 6 P, Peck E, Freeman T, Posaner R. Managing across diverse networks of care: Lessons from other sectors. Report to the National Co-ordinating Centre for the NHS Service Delivery and Organisation R&D. London, Co-ordinating Centre for NHS Delivery and Organisation. 2004. http://www.netscc.ac.uk/hsdr/files/adhoc/39-policy-report.pdf. Accessed 23 October 2016.

[14] Plastrik P and Taylor M. NET GAINS: A Handbook for Network Builders Seeking Social Change Version 1.0. 2006. https://networkimpact.org/downloads/NetGainsHandbookVersion1.pdf. Accessed 23 Oct 2016.

[15] West E, Barron DN, Dowsett J, Newton JN (1999). Hierarchies and cliques in the social networls of health care professionals: implications fort he design of dissemination strategies. Soc Sci Med.

[16] Prats MX (2015). Citation of the opening speech of the second conference on ERN of the EU-Commission in Lisbon on the 8^th^ of.

[17] Nonaka I, Takeuchi H. The Knowledge-Creating Company: How Japanese Companies Create the Dynamics of Innovation. New York: Oxford University Press; 1995.

[18] Gray M. Expessed in a personal interview on the 14th of October 2015.

[19] NHS Leadership Academy. Leadership framework – a summary. 2011. http://www.leadershipacademy.nhs.uk/wp-content/uploads/2012/11/NHSLeadership-Framework-LeadershipFramework-Summary.pdf. Accessed 30 August 2016.

[20] Pelletier D, Wildhaber F, Collerette P, Heberer M (2014). Management, structure and perceived outcomes of hospital alliances: an exploratory multinational study. Universal Journal of Public Health.

[21] NHS Service Delivery and Organisation R&D Programme. Key lessons for network management in health care published by the National Coordinating Centre for Service Delivery and Organisation (NCCSDO) Southampton. 2004. http://www.netscc.ac.uk/hsdr/files/project/SDO_BP_08-1218-039_V01.pdf. Accessed 23 Oct 2016.

[22] Lega F (2005). Strategies for multihospital networks: a framework. Health Serv Manag Res.

[23] Michelis D, Schildhauer T (2010). Social media Handbuch. Theorien, Methoden, Modelle und praxis.

[24] Siemens G. Connectivism: A Learning Theory for the Digital Age. International Journal of Instructional Technology and Distance Learning. 2005. Vol. 2 No. 1. ISSN 1550–6908.

[25] Vicari S, Cappai F. Health activism and the logic of connective action. A case study of rare disease patient organisations.Information, Communication & Society published by Taylor and Francis, ISSN 1468–4462. 2016. 10.1080/1369118X.2016.1154587 PMC4959124 27499676

[26] Douzgou S, Pollalis YA, Vozikis A, Patrinos GP, Clayton-Smith J (2016). Collaborative Crowdsourcing for the diagnosis of rare genetic syndromes: the DYSCERNE experience. Public Health Genomics.

[27] Ranard BL, Ha YP, Meisel ZF, Asch DA, Hill SS, Becker LB, Seymour AK, Merchant RM: Crowdsourcing – harnessing the masses to advance health and medicine, a systematic review. J Gen Intern Med. 2014;29(1):187–203. doi:10.1007/s11606-013-2536-8. Epub 2013 Jul 11. 10.1007/s11606-013-2536-8 PMC3889976 23843021

[28] https://arztkonsultation.de/about. Accessed 3 Mar 2017.

[29] Sheaff R, Benson L, Farbus L, Schofield J, Mannion R, Reeves D (2010). Network resilience in the face of health system reform. Soc Sci Med.

[30] Hollak C, Biegstraaten M, Baumgartner M, Belmatoug N, Bembi B, Bosch A, Brouwers M, Dekker H, Dobbelaere D, Engelen M, Groenendijk M, Lachmann R, Langendonk J, Langeveld M, Linthorst G, Morava E, Poll-The B, Rahman S, Rubio-Gozalbo M, Spiekerkoetter U, Treacy E, Wanders R, Zschocke J, Hagendijk R (2016). Position statement on the role of healthcare professionals, patient organizations and industry in European Reference networks. Orphanet Journal of Rare Diseases.

